# Integrating Typhoid Fever Within the Sustainable Development Goals: Pragmatism or Utopia?

**DOI:** 10.1093/cid/ciy957

**Published:** 2019-02-15

**Authors:** Zulfiqar A Bhutta

**Affiliations:** Centre for Global Child Health, The Hospital for Sick Children, Toronto, Ontario, Canada

## Abstract

Several decades following the first estimates of the global burden of typhoidal salmonellosis (infections caused by *Salmonella* Typhi and *Salmonella* Paratyphi), this disorder remains a major cause of morbidity worldwide with an estimated 17 million cases annually. The risk factors for typhoid include poverty, poor living conditions with unsafe water and lack of adequate sanitation, and unsafe foods—all reasons for the disease burden being highest among such populations including urban slums. A recent review of typhoid trends globally and in specific countries suggests that the relative contributions of these risk factors to disease burden reduction as well as persistence have varied. There is also the risk of periodic outbreaks related to introduction of relatively virulent drug-resistant strains or movements of vulnerable populations, including those in conflict zones. Most countries of the world are now aligning their health and multisectoral strategies to address the Sustainable Development Goals (SDGs) and targets, which were agreed upon by all countries of the world in September 2015. Though neglected so far, there are huge opportunities for mainstreaming typhoid prevention and control strategies within the SDGs. This article reviews some of the approaches that may help elevate typhoid to a higher level of awareness in public health programs and policy and to ensure that investments in major public health preventive measures are made part of the universal health coverage agenda.

Although exact numbers are difficult to estimate in the absence of robust surveillance systems, an estimated 17 million cases of typhoid and paratyphoid fever illnesses (typhoidal salmonellosis, hereafter called typhoid for simplicity) occurred globally in 2015 [1] mostly in South Asia, Southeast Asia, and sub-Saharan Africa, with the largest burden estimated in South Asia [2]. More data regarding the incidence of typhoid fever in Africa has been generated in recent years including the recently published Typhoid Fever Surveillance in Africa Program supplement, although additional challenges to estimating the incidence of typhoid fever accurately remain prominent [3, 4]. Despite available antibiotic therapy, both typhoid and paratyphoid fever can lead to serious illnesses with an estimated 178 000 deaths globally in 2015 [1, 5]. With emergence of antibiotic resistance, the morbidity and severity of illness may be higher in drug-resistant cases of typhoid and has been noted to be a major cause of concern, and an important consideration behind the recent SAGE recommendation for introduction of typhoid conjugate vaccination in endemic countries [5]. While the introduction of typhoid conjugate vaccines and the willingness of global support mechanisms through the Gavi funding mechanisms are important strategies, typhoid control merits a wider strategy as an important adjunct to prevention strategies. A recent review of implementation of known typhoid fever preventive strategies in 8 target countries revealed major gaps and variability in coverage, with opportunities for scaling up [6].

## RISK FACTORS AND DETERMINANTS OF TYPHOID

Typhoid was a virtually invisible disease during the period of the Millennium Development Goals (MDGs) from 2000 to 2015, a period that saw much global action around maternal and child health [7]. There were several reasons for this, including a major focus on maternal and child mortality and a rather limited attention to the social determinants of health. Much of this has changed with the opportunity provided by the Sustainable Development Goals (SDGs), which were approved in 2015 after an extensive process of stakeholder and country consultation [8]. The SDGs encompass many goals that relate to environment, risk factors, and underlying determinants of disease; several of these are particularly germane to typhoid.

Table 1 summarizes some of the known risk factors for typhoid fever outbreaks from recent studies assessing such risks [9–21], including elements related to socioeconomic deprivation, poor water and sanitation, and food safety. Additionally, health services and system matter a great deal, both in terms of access to appropriate diagnostic and curative services as well as the vaccination strategies. This is especially notable with the continued uncertainty around the burden and severity of typhoid, even though cases of typhoid complications such as perforations still abound [22].

**Table 1.** Risk Factors for Typhoid From Relatively Recent Studies

Study	Country	Drinking Water	Hand Washing	Open Defecation	Consumption of Raw Vegetables/Fruits	Street Foods	Household Size	Literacy Rates	Use of Antibiotics 2 wk Prior
Sur et al, 2007 ^[9]^	India						✓	✓	
Alba et al, 2016 ^[10]^	Indonesia		✓			✓			
Hosoglu et al, 2006 ^[11]^	Turkey				✓				
Bhunia et al, 2009 ^[12]^	India	✓			✓				
Khan et al, 2012 ^[13]^	Pakistan	✓					✓		
Ram et al, 2007 ^[14]^	Bangladesh	✓		✓	✓				
Srikantiah et al, 2007 ^[15]^	Uzbekistan	✓							✓
Gasem et al, 2001 ^[16]^	Indonesia	✓	✓			✓			
Sharma et al, 2009 ^[17]^	India	✓		✓	✓				
Tran et al, 2005 ^[18]^	Vietnam	✓							
Vollaard et al, 2005 ^[19]^	Indonesia		✓			✓			
Kabwama et al, 2017 ^[20]^	Uganda	✓							
Barac et al, 2018 ^[6]^	Chile, India, Pakistan, Bangladesh, Thailand, Vietnam, South Africa, and Nigeria	✓	✓	✓		✓	✓	✓	

## DO THE SUSTAINABLE DEVELOPMENT GOALS OFFER AN OPPORTUNITY FOR ADDRESSING TYPHOID AT SCALE?

The SDGs are the blueprint to achieve a better and more sustainable future for all. They address most of the global challenges we face, including those related to poverty, inequality, climate, environmental degradation, prosperity, and peace and justice. The Goals are interconnected and differ from the previous MDGs in that they are wide ranging and relate to global priorities for the next generation. There is also an explicit focus on reducing inequalities and reaching marginalized populations. While the 17 SDGs, 230 individual indicators, and 169 associated targets may seem impossibly wide-ranging and aspirational, they are interrelated and overall progress focuses on multisectoral approaches [23]. Health is allocated just one goal on its own (Goal 3, to ensure healthy lives and promote well-being for all at all ages), but many of the other SDGs relate to the social determinants of health and are interconnected [24]. There are ample reasons for addressing the prevention and management strategies for typhoid as part of a global action plan or package for countries with residual burden or high-risk burden of disease. These include many low- and middle-income countries (LMICs) with populations at risk, frequently among urban slums and displaced populations. In other cases, including high-income settings, typhoid may relate to travel to endemic areas and imported cases through visitors or migrant workers. Factors such as crowding, poor sanitation, and unsafe agricultural or food production processes also contribute to typhoid burden and transmission risks. Therefore, measures to interrupt transmission through improvements in sanitation and food production need to be included in comprehensive prevention strategies for enteric fever.

Many of the SDGs and related indicators directly and indirectly relate to typhoid prevention, control, and management. Table 2 summarizes the relevant SDGs, relevant indicators, and elements related to typhoid. These risks are compounded by poor diagnostic facilities and quality of care within health systems, leading to delay in the institution of appropriate therapy. In those LMICs where disease burden remains high, strategies to interrupt typhoid transmission must target health systems in an attempt to improve on typhoid prevention and treatment. The emergence of drug-resistant strains of typhoid has posed additional challenges in the management and containment of disease [25, 26].

**Table 2.** Summary Table of Sustainable Development Goal Indicators of Relevance to Typhoid and Its Control

Target	Relevant Indicator	Relevance to Typhoid Prevention and Control
Goal 1. End poverty in all its forms everywhere			
1.1 By 2030, eradicate extreme poverty for all people everywhere, currently measured as people living on less than $1.25 a day	1.1.1 Proportion of population below the international poverty line, by sex, age, employment status, and geographical location (urban/rural)	**Overall poverty levels and its clustering especially among its slums; poor populations are major risk factors for enteric infections and typhoid**
1.2 By 2030, reduce at least by half the proportion of men, women, and children of all ages living in poverty in all its dimensions according to national definitions	1.2.1 Proportion of population living below the national poverty line, by sex and age	
	1.2.2 Proportion of men, women, and children of all ages living in poverty in all its dimensions according to national definitions	
1.3 Implement nationally appropriate social protection systems and measures for all, including floors, and by 2030 achieve substantial coverage of the poor and the vulnerable	1.3.1 Proportion of population covered by social protection floors/systems, by sex, distinguishing children, unemployed persons, older persons, persons with disabilities, pregnant women, newborns, work-injury victims, and the poor and the vulnerable	Access to social protection services and safety nets are important in typhoid control (through early care seeking and provision of services)
1.4 By 2030, ensure that all men and women, in particular the poor and the vulnerable, have equal rights to economic resources, as well as access to basic services, ownership, and control over land and other forms of property, inheritance, natural resources, appropriate new technology, and financial services, including microfinance	1.4.1 Proportion of population living in households with access to basic services	
1.A Ensure significant mobilization of resources from a variety of sources, including through enhanced development cooperation, in order to provide adequate and predictable means for developing countries, in particular least-developed countries, to implement programs and policies to end poverty in all its dimensions	1.A.1 Proportion of domestically generated resources allocated by the government directly to poverty reduction programs	Critical national investments for addressing social determinants of health and making healthcare affordable and accessible for those below the poverty line. Could include safety nets and voucher schemes to address this as well as health insurance schemes.
	1.A.2 Proportion of total government spending on essential services (education, health, and social protection)	
1.B Create sound policy frameworks at the national, regional, and international levels, based on pro-poor and gender-sensitive development strategies, to support accelerated investment in poverty eradication actions	1.B.1 Proportion of government recurrent and capital spending to sectors that disproportionately benefit women, the poor, and vulnerable groups	Given that poverty has clear gender dimensions, such strategies should specifically target poor women including heads of households
Goal 2. End hunger, achieve food security and improved nutrition, and promote sustainable agriculture		
2.1 By 2030, end hunger and ensure access by all people, in particular the poor and people in vulnerable situations, including infants, to safe, nutritious, and sufficient food all year round	2.1.1 Prevalence of undernourishment	Malnutrition is known to increase the risks of morbidity and adverse outcomes associated with typhoid. **While strategies to address food security are important, given known risk with unsafe foods, especially street foods, food safety in endemic countries.**
	2.1.2 Prevalence of moderate or severe food insecurity in the population, based on the Food Insecurity Experience Scale	
2.2 By 2030, end all forms of malnutrition, including achieving, by 2025, the internationally agreed targets on stunting and wasting in children <5 years of age, and address the nutritional needs of adolescent girls, pregnant and lactating women, and older persons	2.2.1 Prevalence of stunting (height for age <–2 SD from the median of the WHO Child Growth Standards) among children <5 years of age	
	2.2.2 Prevalence of malnutrition (weight for height >+2 or <–2 SD from the median of the WHO Child Growth Standards) among children <5 years of age, by type (wasting and overweight)	
Goal 3. Ensure healthy lives and promote well-being for all at all ages		
	3.3.5 Number of people requiring interventions against neglected tropical diseases	
3.8 Achieve universal health coverage, including financial risk protection, access to quality essential healthcare services, and access to safe, effective, quality, and affordable essential medicines and vaccines for all	3.8.1 Coverage of essential health services (defined as the average coverage of essential services based on tracer interventions that include reproductive, maternal, newborn and child health, infectious diseases, noncommunicable diseases, and service capacity and access, among the general and the most disadvantaged population)	Catastrophic health expenditures on treatment of the disease and addressing complications of typhoid, especially among drug-resistant infections, are well recognized. This is an important consideration within health systems and in strategies from governments to address universal health coverage, a key target for the SDGs.
	3.8.2 Proportion of population with large household expenditures on health as a share of total household expenditure or income	
3.9 By 2030, substantially reduce the number of deaths and illnesses from hazardous chemicals and air, water, and soil pollution and contamination	3.9.2 Mortality rate attributed to unsafe water, unsafe sanitation, and lack of hygiene (exposure to unsafe WASH services)	**Addressing access to safe water and reduction of unsafe sanitation is critical to reduction of diarrheal disease burdens and reduction of typhoid risk**
3.B Support the research and development of vaccines and medicines for the communicable and noncommunicable diseases that primarily affect developing countries, provide access to affordable essential medicines and vaccines, in accordance with the Doha Declaration on the TRIPS Agreement and Public Health, which affirms the right of developing countries to use to the full the provisions in the Agreement on Trade-Related Aspects of Intellectual Property Rights regarding flexibilities to protect public health, and, in particular, provide access to medicines for all	3.B.1 Proportion of the target population covered by all vaccines included in their national program	**The recent introduction of typhoid conjugate vaccines offers huge opportunities for protecting at-risk populations expeditiously through targeted campaigns and integration within EPI strategies in high-burden countries**
	3.B.2 Total net official development assistance to medical research and basic health sectors	Every country ought to invest in appropriate research (epidemiological studies, addressing risk factors and implementation research) to address typhoid and other enteric infections
	3.B.3 Proportion of health facilities that have a core set of relevant essential medicines available and affordable on a sustainable basis	**Availability of appropriate diagnostics and antibiotics for typhoid (both first- and second-line) are a cornerstone for typhoid management and control**
3.C Substantially increase health financing and the recruitment, development, training, and retention of the health workforce in developing countries, especially in least-developed countries and small island developing states	3.C.1 Health worker density and distribution	None of the above is possible without trained and adequate health personnel and is clearly linked with national human resource policies, training of staff, and appropriate staffing patterns/distribution
3.D Strengthen the capacity of all countries, in particular developing countries, for early warning, risk reduction, and management of national and global health risks	3.D.1 International Health Regulations capacity and health emergency preparedness	Although typhoid is a notifiable disease, this is only adequately implemented in a handful of countries. As drug-resistant strains of typhoid proliferate, attention must be given to travelers to and from endemic areas in terms of vaccination coverage and follow-up.
Goal 5. Achieve gender equality and empower all women and girls		
5.C Adopt and strengthen sound policies and enforceable legislation for the promotion of gender equality and the empowerment of all women and girls at all levels	5.C.1 Proportion of countries with systems to track and make public allocations for gender equality and women’s empowerment	While not directly related to typhoid control, the known gender disparities in healthcare and care-seeking patterns merit attention to the gender dimensions of typhoid from an early stage.
Goal 6. Ensure availability and sustainable management of water and sanitation for all		
6.1 By 2030, achieve universal and equitable access to safe and affordable drinking water for all	6.1.1 Proportion of population using safely managed drinking water services	Safe water and sanitation services are a critical investment for addressing common risks for typhoid and other enteric infections, enteropathy, and malnutrition and should be scaled up. **The focus should change from mere improved water to safe water and elimination of open defecation in all countries of the world.** This is especially important in water-insecure environments affected by climate change and rapid urbanization.
6.2 By 2030, achieve access to adequate and equitable sanitation and hygiene for all and end open defecation, paying special attention to the needs of women and girls and those in vulnerable situations	6.2.1 Proportion of population using safely managed sanitation services, including a hand-washing facility with soap and water	
6.3 By 2030, improve water quality by reducing pollution, eliminating dumping, and minimizing release of hazardous chemicals and materials, halving the proportion of untreated wastewater and substantially increasing recycling and safe reuse globally	6.3.1 Proportion of wastewater safely treated	**This is an essential intervention alongside provision of safe water and sanitary measures.**
	6.3.2 Proportion of bodies of water with good ambient water quality	
6.A By 2030, expand international cooperation and capacity-building support to developing countries in water- and sanitation-related activities and programs, including water harvesting, desalination, water efficiency, wastewater treatment, recycling, and reuse technologies	6.A.1 Amount of water- and sanitation-related official development assistance that is part of a government-coordinated spending plan	Reflects community ownership of measures to improve WASH-related investments, which, in addition to the clear health and nutrition benefits, should be instituted on the basis of fundamental human rights considerations
6.B Support and strengthen the participation of local communities in improving water and sanitation management	6.B.1 Proportion of local administrative units with established and operational policies and procedures for participation of local communities in water and sanitation management	
Goal 8. Promote sustained, inclusive, and sustainable economic growth; full and productive employment; and decent work for all		
8.1 Sustain per capita economic growth in accordance with national circumstances and, in particular, at least 7% GDP growth per annum in the least-developed countries	8.1.1 Annual growth rate of real GDP per capita	A key factor responsible for sustainable development and national investments for which economic growth is important
Goal 9. Build resilient infrastructure, promote inclusive and sustainable industrialization, and foster innovation		
9.1 Develop quality, reliable, sustainable, and resilient infrastructure, including regional and transborder infrastructure, to support economic development and human well-being, with a focus on affordable and equitable access for all	9.1.1 Proportion of the rural population who live within 2 km of an all-season road	Access and communication strategies are an essential part of national health system and a prerequisite for development
	9.1.2 Passenger and freight volumes, by mode of transport	
9.5 Enhance scientific research, upgrade the technological capabilities of industrial sectors in all countries (in particular developing countries), including, by 2030, encouraging innovation and substantially increasing the number of research and development workers per 1 million people and public and private research and development spending	9.5.1 Research and development expenditure as a proportion of GDP	**Essential national research into disease burden and resistance patterns are an important part of national response to the challenges of infectious diseases and amenable mortality and morbidity. Given the rise in super-resistant strains of typhoid, this is a priority for typhoid-endemic countries.**
	9.5.2 Researchers (in full-time equivalent) per million inhabitants	
Goal 10. Reduce inequality within and among countries		
10.1 By 2030, progressively achieve and sustain income growth of the bottom 40% of the population at a rate higher than the national average	10.1.1 Growth rates of household expenditure or income per capita among the bottom 40% of the population and the total population	Essential for equitable development and economic growth within countries
Goal 11. Make cities and human settlements inclusive, safe, resilient, and sustainable		
11.1 By 2030, ensure access for all to adequate, safe, and affordable housing and basic services and upgrade slums	11.1.1 Proportion of urban population living in slums, informal settlements, or inadequate housing	**This is especially relevant to megacities with large slum populations and recent migrants, hot spots for disease transmission and outbreaks, including typhoid.** Among issues such as living conditions, population density per household, lack of adequate sanitation facilities, and waste disposal play a major role in continued typhoid burden and transmission
11.6 By 2030, reduce the adverse per capita environmental impact of cities, including by paying special attention to air quality and municipal and other waste management	11.6.1 Proportion of urban solid waste regularly collected and with adequate final discharge out of total urban solid waste generated by cities	
Goal 12. Ensure sustainable consumption and production patterns		
12.4 By 2020, achieve the environmentally sound management of chemicals and all wastes throughout their life cycle, in accordance with agreed international frameworks, and significantly reduce their release to air, water, and soil in order to minimize their adverse impacts on human health and the environment	12.4.1 Number of parties to international multilateral environmental agreements on hazardous waste, and other chemicals that meet their commitments and obligations in transmitting information as required by each relevant agreement	Relevant to human and hospital waste management
	12.4.2 Hazardous waste generated per capita and proportion of hazardous waste treated, by type of treatment	
Goal 16. Promote peaceful and inclusive societies for sustainable development, provide access to justice for all, and build effective, accountable, and inclusive institutions at all levels		
16.5 Substantially reduce corruption and bribery in all their forms	16.5.1 Proportion of persons who had at least one contact with a public official and who paid a bribe to a public official, or were asked for a bribe by those public officials, during the previous 12 months	Recognized as an important factor related to dysfunction and poor management at various levels of the health system and in a disease like typhoid, important at various levels related to procurements and quality of care
	16.5.2 Proportion of businesses that had at least one contact with a public official and that paid a bribe to a public official, or were asked for a bribe by those public officials during the previous 12 months	
Goal 17. Strengthen the means of implementation and revitalize the Global Partnership for Sustainable Development		
Finance		
17.1 Strengthen domestic resource mobilization, including through international support to developing countries, to improve domestic capacity for tax and other revenue collection	17.1.1 Total government revenue as a proportion of GDP, by source	An important initiative to secure enhanced domestic financing for health, especially given recent evidence that during the Millennium Development Goal period, external assistance frequently led to displacement of financing and reduced proportional national spending on health
	17.1.2 Proportion of domestic budget funded by domestic taxes	
17.2 Developed countries to implement fully their official development assistance commitments, including the commitment by many developed countries to achieve the target of 0.7% of ODA/GNI to developing countries and 0.15–0.20% of ODA/GNI to least-developed countries; ODA providers are encouraged to consider setting a target to provide at least 0.20% of ODA/GNI to least developed countries	17.2.1 Net official development assistance, total and to least-developed countries, as a proportion of the OECD Development Assistance Committee donors’ GNI	To the most impoverished and indebted countries, this is an important initiative. **The recent global financing facility for women and children’s health as well as the Gavi assistance window for typhoid conjugate vaccination also offers additional incentives for supporting typhoid-specific initiatives in countries.**
Capacity-building		
17.9 Enhance international support for implementing effective and targeted capacity-building in developing countries to support national plans to implement all the SDGs, including through North–South, South–South, and triangular cooperation	17.9.1 Dollar value of financial and technical assistance (including through North–South, South–South, and triangular cooperation) committed to developing countries	These human resources include all ranges of expertise needed at country level including those engaged in implementation, planning and execution, and monitoring and evaluation.
Data, monitoring and accountability		
17.18 By 2020, enhance capacity-building support to developing countries, including for least-developed countries and small island developing states, to increase significantly the availability of high-quality, timely, and reliable data disaggregated by income, gender, age, race, ethnicity, migratory status, disability, geographic location, and other characteristics relevant in national contexts	17.18.1 Proportion of sustainable development indicators produced at the national level with full disaggregation when relevant to the target, in accordance with the Fundamental Principles of Official Statistics	These broad measures to collect information on key indicators for the SDGs, and especially those that are related to health, offer special opportunities for collecting information on typhoid fever through routine information systems. **While few countries have established robust surveillance systems for typhoid, these are possible to integrate within district-based health information systems with a minimal set of data on typhoid admissions, antimicrobial resistance patterns among proven cases, and complications** (perforations, deaths, etc)
	17.19.2 Proportion of countries that (a) have conducted at least one population and housing census in the last 10 years; and (b) have achieved 100% birth registration and 80% death registration	

Bold text indicates items that are especially relevant to integrated typhoid control strategies.

Abbreviations: EPI, Expanded Programme on Immunization; GDP, gross domestic product; GNI, gross national income; ODA, official development assistance; OECD, Organization for Economic Cooperation and Development; SD, standard deviation; SDG, Sustainable Development Goals; TRIPS, xxx; WASH, water, sanitation, and hygiene; WHO, World Health Organization.

An analysis of typhoid and paratyphoid fever management in India found that the average cost per case treated was approximately US$53, which is potentially unaffordable in many high-burden regions [27]. Courses of second- and third-line antibiotic therapy for newer quinolone and now cephalosporin-resistant strains of typhoid are even more expensive and frequently not available within the public sector. In the absence of safety nets for typhoid prevention and management, one can envision that a single case of multidrug-resistant typhoid could be a catastrophic illness pushing some over the poverty line. With the focus on universal health coverage within the SDG 3 for health, a major challenge will be provision of services for those presenting with undiagnosed febrile illnesses (including dengue, typhoid, malaria, and chikungunya), where rapid diagnostics understandably hold the key for appropriate triage and therapy [28].

## HOW DO WE MONITOR PROGRESS AND ACCOUNTABILITY?

A major problem in global typhoid monitoring and control is lack of adequate data and information on burden and trends. That is probably one of the reasons as to why the disorder was virtually orphaned between various departments at the World Health Organization. Despite the fact that the bulk of the disease is in the pediatric population, this was never a priority within the Child and Adolescent Health Department and was not included among the IMCI regimen dealing with febrile illnesses. Typhoid was not accorded the category of a neglected tropical disease either, and was relegated to the Vaccines and Biologicals program for development of global guidance, which led to a position paper in 2003 [29], which has only just been updated as a position paper on typhoid vaccines [30]. Although better information is now available from special studies and research programs, a review of the global burden by Crump et al showed that from 1954 to 2000, only 13 countries had population-based surveillance data for typhoid, and only 2 countries within Africa (Egypt and South Africa) had surveillance data from the control arms of vaccination trials [31]. To effectively allocate resources for complex interventions, it is essential to have accurate knowledge about local disease burden, risk factors, and locally appropriate control measures. A case can thus be made for developing and implementing robust surveillance systems for typhoid.

## A FRESH HOPE FOR PREVENTION

Typhoid progress has for long been hampered by the lack of an effective vaccine, especially among the under-5 population. The existing Vi polysaccharide vaccine, even though effective in older children and adults, has waning immunity after a few years and an overall protective efficacy of 69% (95% confidence interval, 63%–74%) [32]. It is also in this context that the introduction of the new typhoid conjugate vaccine, with enhanced efficacy against typhoid among young children, offers an extremely important adjunct to existing tools for the prevention of typhoid and potentially averting the emergence of super-resistant strains of typhoid [33]. The vaccination strategies offer opportunities for enhancing protective population immunity for those at risk through targeted vaccination campaigns and potential integration of typhoid vaccines into childhood immunization schedules.

## CONCLUSIONS

Countries need to invest in a comprehensive strategy for typhoid control that addresses key determinants and risk factors for the disease. In many developing countries, significant parts of the population reside in places where a substantial proportion of the population lacks access to improved drinking water and sanitation, and live in congested slums with conditions favorable for the transmission of typhoid fever and other enteric pathogens. It is recognized that improvements in water supply and sanitation infrastructure, as well as food safety legislation, effected dramatic reductions in typhoid incidence in most of Europe and the United States [22], with the residual disease burden largely related to travelers returning from countries where typhoid fever remains endemic. In addition, a focus must be placed on individual behaviors and water, sanitation, and hygiene infrastructure. These risk factors are compounded by poor regulatory environments and poor food safety with potential access to contaminated foods. These factors contribute to relatively excess risks of typhoid and high burden of disease among young children and adolescents [34]. Given differing risks of transmission and force of infection, despite marked reductions in diarrheal disease burdens, many countries in South Asia and sub-Saharan Africa still continue to have a significant burden of typhoid.

Urgent action is needed in the context of integrating typhoid within the SDGs. It is important to underscore that, while typhoid remains a global public health challenge, it has not, as yet, risen to a level of priority needing concerted action (and financing), and the SDGs offer a unique opportunity to address this. This requires a multipronged strategy to raise awareness at the national, regional, and global levels on the importance of typhoid as a preventable disease. This includes continued investments in reducing risks associated with poverty, poor living conditions, and poor sanitation; continued investments in safe water and foods; initiatives to improve access, rapid diagnosis, and care; and targeted immunization strategies within health systems [35]. The recent spread of antimicrobial resistance and global support for initiatives to mitigate such risks offer a huge opportunity for rationalizing diagnosis and treatment and preventive approaches. This would mean including typhoid-related estimates and outcomes among the universal health coverage–related strategies and monitoring that countries are drawing up presently.

## Notes

***Financial support.*** Z. A. B. has received a grant from the Bill & Melinda Gates Foundation.

***Supplement sponsorship.*** This supplement was sponsored by Coalition against Typhoid, Sabin Vaccine Institute, Bill & Melinda Gates Foundation.

***Potential conflicts of interest.*** Author certifies no reported conflicts of interest. The author has submitted the ICMJE Form for Disclosure of Potential Conflicts of Interest. Conflicts that the editors consider relevant to the content of the manuscript have been disclosed.
